# Supplementary material for: Tead1a Initiates Transcriptional Priming Through the TEAD1a/YAP‐Notch1‐Spi1/Cebpα Axis to Promote Neutrophil Fate
Source: Adv Sci (Weinh). 2025 Aug 28;12(41):e05441. doi: 10.1002/advs.202505441 (PMC12591127; doi:10.1002/advs.202505441)
Supplement: Supplementary file 4 — Supplementary Dataset [file ADVS-12-e05441-s004.zip › 3-6.zip!/3-6/02-6.cluster/cluster15/Enrich/ego_MF.pdf]

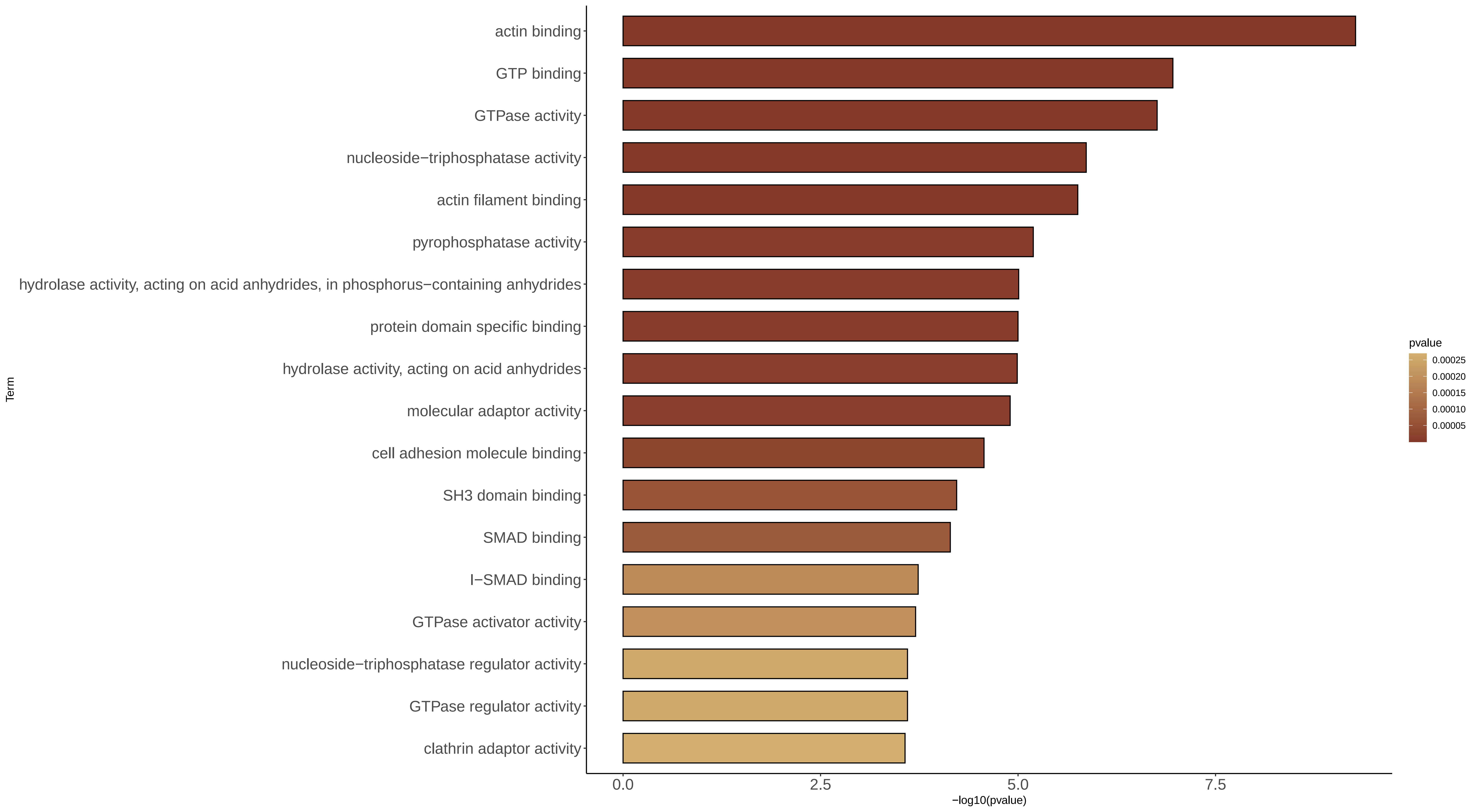

actin binding
GTP binding
GTPase activity
nucleoside−triphosphatase activity
actin filament binding
pyrophosphatase activity
hydrolase activity, acting on acid anhydrides, in phosphorus−containing anhydrides
protein domain specific binding
hydrolase activity, acting on acid anhydrides
molecular adaptor activity
cell adhesion molecule binding
SH3 domain binding
SMAD binding
I−SMAD binding
GTPase activator activity
nucleoside−triphosphatase regulator activity
GTPase regulator activity
clathrin adaptor activity
Term
0.0
2.5
5.0
7.5
−log10(pvalue)
pvalue
0.00025
0.00020
0.00015
0.00010
0.00005
